# Characteristics of late-onset spondyloarthritis in Japan: A retrospective cohort study

**DOI:** 10.1097/MD.0000000000014431

**Published:** 2019-02-15

**Authors:** Yushiro Endo, Keita Fujikawa, Tomohiro Koga, Akinari Mizokami, Masanobu Mine, Toshiaki Tsukada, Masataka Uetani, Atsushi Kawakami

**Affiliations:** a Department of Rheumatology, Japan Community Healthcare Organization, Isahaya General Hospital; b Department of Immunology and Rheumatology, Unit of Advanced Preventive Medical Sciences, Nagasaki University Graduate School of Biomedical Sciences; c Department of Rheumatology, Suga Orthopedic Hospital; d Department of Rheumatology, Aino Memorial Hospital; e Department of Radiological Sciences, Graduate School of Biomedical Sciences, Nagasaki University, Japan.

**Keywords:** early-onset spondyloarthritis, late-onset spondyloarthritis, peripheral spondyloarthritis, spondyloarthritis

## Abstract

Spondyloarthritis may be increasingly present in older patients as life expectancy increases. We investigated clinical differences between early-onset and late-onset spondyloarthritis in Japan.

We retrospectively reviewed 114 patients consecutively diagnosed with spondyloarthritis. The clinical course of each patient was observed for ≥1 year. We defined early-onset and late-onset spondyloarthritis as <57 or ≥57 years at a median age of this study group, respectively. We compared clinical characteristics between these 2 groups.

Disease duration was significantly shorter before diagnosis in the late-onset group (*P* < .01). Inflammatory back pain (IBP) was significantly more common in the early-onset group (*P* < .01), whereas dactylitis frequency was significantly higher in the late-onset group. Significantly more patients with early-onset spondyloarthritis were human leukocyte antigen (HLA) B27-positive (*P* < .01). Articular synovitis, particularly of the wrist, was significantly more common on power Doppler ultrasound (PDUS) in the late-onset group (*P* < .01). Tenosynovitis or peritendinitis, particularly in the finger and wrist flexors were also more frequent in the late-onset group (*P* < .001 and *P* < .05, respectively). Enthesitis of the finger collateral ligament and lateral collateral ligament were significantly more common in the late-onset group (both *P* < .05). Multiple logistic regression analysis revealed that, comparatively, IBP was significantly and independently much more likely to occur in the early-onset group.

The patients with late-onset spondyloarthritis had a lower frequency of IBP and HLA B27 and a higher frequency of dactylitis and PDUS findings in peripheral involvement.

## 1 Introduction

Spondyloarthritis (SpA) diseases share similar clinical manifestations, such as involvement of sacroiliac joints, spine, peripheral joints, and skin as well as mucosal involvement. In the 1970s, Moll and Wright established the concept of a group of interrelated disorders termed seronegative spondyloarthritides.^[1]^ The European Spondylarthropathy Study Group (ESSG) established the ESSG criteria for a group of diseases known as SpA, which included ankylosing spondylitis (AS), psoriatic arthritis (PsA), inflammatory bowel disease (IBD)-related arthritis, reactive arthritis (ReA), and undifferentiated SpA (uSpA), an entity that does not fit in any of the other classifications.^[2,3]^ Additionally, the assessment of SpondyloArthritis International Society (ASAS) grouped SpA into 2 categories based on the predominant clinical presentation: axial SpA (axSpA) or peripheral SpA (pSpA).^[4–6]^ Although conventional radiography reflects the structural consequences of the inflammatory process, magnetic resonance imaging (MRI) can detect early inflammatory changes before the appearance of sacroiliitis on X-ray.^[7,8]^ Non-radiographic axSpA developed by the ASAS is the term used for an axSpA without sacroiliitis seen on X-ray.^[5]^

The frequency of human leukocyte antigen (HLA) B27-positivity, which is associated with SpA, varies substantially among individuals of different countries.^[9]^ In Japan, the prevalence of HLA B27 is 0.3% in the general population,^[10]^ which is much lower than that in other countries. However, the frequency of HLA B27-positivity in Japanese SpA patients has not been clarified.

Onset of SpA in older adults has been considered rare,^[11]^ but cases are increasingly reported.^[12–14]^ The number of such patients is expected to increase with longer life expectancy.^[11,13–16]^ Later- or late-onset SpA (LOSpA)^[11,13,15,16]^ has not been precisely defined, but most studies have used onset at 50 years or older to classify such patients.^[13]^ Previous studies that assessed areas with high HLA B27 prevalence revealed differences between characteristics of early-onset SpA (EOSpA) and LOSpA.^[11,14,15]^ However, the findings in LOSpA have not yet been reported for Japan, a super-aging society.

In the present study, we compared the clinical profiles of patients with EOSpA or LOSpA to describe the clinical characteristics of LOSpA in Japan.

## 2 Materials and methods

### 2.1 Patients

This was a single-center retrospective cohort study approved by the medical ethics committee of Japan Community Healthcare Organization Isahaya General Hospital. The study population consisted of 114 Japanese patients with SpA who were managed between April 2009 and December 2017 at our institution. The present study included both incident and prevalent cases. The clinical course of each patient was observed for ≥1 year. A definitive diagnosis of SpA was made by a Japan College of Rheumatology (JCR)-certified rheumatologist (KF, AM, MM, and TT) after excluding other rheumatic diseases by evaluating the following items: medical history, physical examination, laboratory findings, imaging findings, SpA classification criteria, and therapeutic response. Inflammatory back pain (IBP) was defined by the Berlin criteria.^[17]^ Enthesis tenderness was evaluated on clinical examination according to the Leeds Enthesis Index^[18]^ and the Spondyloarthritis Research Consortium of Canada.^[19]^ The inclusion criteria were any of the various sets of SpA classification criteria that include the following: Amor,^[2]^ ESSG,^[3]^ ASAS criteria for axSpA,^[5]^ ASAS criteria for pSpA,^[6]^ and modified New York^[20]^ criteria. Thus, all patients satisfied 1 or more of the SpA classification criteria. We defined radiographic sacroiliitis as grade ≥2 bilaterally or grade 3-4 unilaterally on pelvic X-ray according to the modified New York criteria.^[20]^ In cases wherein radiographic sacroiliitis was uncertain or there was persistent IBP, MRI of the SIJs was performed. This was defined in the MRI as the presence of active inflammatory lesions such as bone marrow edema or osteitis as proposed by the ASAS MRI working group.^[21]^ An expert radiologist (MU) interpreted the MRIs. We also performed power Doppler ultrasound (PDUS) assessments in patients with peripheral symptoms. These assessments were done by JCR-registered sonographers (YE, KF). Systematic multiplanar gray scale (GS) and power Doppler (PD) examinations of joints, tendons, and entheses were performed with a LOGIQ S8 ultrasound machine (GE Healthcare, Wauwatosa, WI) using a multifrequency linear transducer (4–15 MHz). We assessed articular synovia by PDUS at the wrists and the metacarpophalangeal (MCP), proximal interphalangeal (PIP), knee, and ankle joints bilaterally. Articular synovitis on PDUS was defined as GS and PD findings in the synovium. Tendons and tendon sheaths were also bilaterally assessed by PDUS at the extensor and flexor tendon sheaths of the wrist, periextensor tendon and flexor tendon sheaths of the finger, and tibialis posterior tendon sheaths. Tenosynovitis and peritendinitis were defined as GS and PD positivity of the tendon sheath or peritendon. Finally, we assessed the entheseal insertions bilaterally with US at the finger collateral ligaments, common extensor tendons on the lateral epicondyle of elbow, quadriceps tendon on the superior pole of the patella, proximal patellar ligament on the inferior pole of patella, distal patellar ligament on the tibial tuberosity, medial collateral ligament on the medial femoral condyle, lateral collateral ligament on the lateral femoral condyle, and Achilles tendon on the calcaneus. Enthesitis was defined on PDUS as a PD signal with or without structural abnormalities of the enthesis. Patients who did not have medical records including onset of disease, HLA-B antigen, and fulfillment of the SpA criteria were excluded. Further, those who did not have a definitive diagnosis and were diagnosed as palmoplantar pustular arthritis and synovitis, acne, pustulosis, hyperostosis, and osteitis (SAPHO) syndrome were excluded.

The definition of age at onset associated with LOSpA has not been consistent among previous reports. In this study, we defined EOSpA and LOSpA as <57 or ≥57 years at a median age of this study group, respectively. We compared clinical manifestations and laboratory and imaging findings between these 2 groups.

### 2.2 Statistical analysis

The demographic and clinical characteristics were compared with Fisher exact test for discrete variables and with Wilcoxon test for continuous variables. The Kruskal–Wallis test followed by Dunn multiple comparisons test was used to compare the 2 groups.

To independently determine factors associated with LOSpA, we performed multiple logistic regression analysis, using variables with *P*-values of <.05 on univariate analysis. Statistical analyses were performed with JMP pro 13.0 software (SAS Institute, Cary, NC). All reported *P*-values are 2-sided. A *P*-value of <.05 was considered statistically significant.

## 3 Results

### 3.1 Patient characteristics and diagnosis

A total of 114 patients including those with EOSpA (n = 55) and LOSpA (n = 59) were reviewed in this study. Among these patients (median age, 57.0 years), 78 (68.4%) were diagnosed as uSpA, 16 (14%) as PsA, 11 (9.7%) as AS, 6 (5.3%) as IBD-related arthritis, 2 (1.8%) as nr-axSpA, and 1 (0.9%) as ReA. The median age at disease onset was 48.0 years for the EOSpA group and 72.0 years for the LOSpA group (Table 1). Disease duration at diagnosis was significantly shorter in the LOSpA group. There was no significant difference in terms of sex and family history of SpA between the 2 groups. As a definitive diagnosis, uSpA was common in both groups, and there was no significant difference in the proportion between the 2 groups.

**Table 1:**
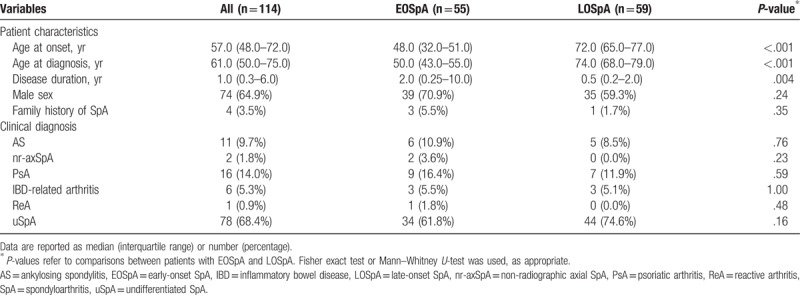
Patient characteristics and clinical diagnosis of patients with EOSpA and LOSpA (univariate analysis).

### 3.2 Clinical features and laboratory characteristics

We found no significant differences in terms of the presence of enthesis tenderness, arthritis of the lower limbs, or uveitis. However, IBP was significantly less common in the LOSpA group, whereas dactylitis was significantly more common in the LOSpA group (Table 2). The RF positivity was 11.4% among all patients, and the average of the RF titer was 28.2 IU/ml (normal range; 0–15 IU/ml) among RF-positive patients with SpA. None of these patients met the 2010 ACR/EULAR classification criteria for rheumatoid arthritis (RA)^[22]^ even after accounting for the number of synovitis in PDUS. The groups did not differ in terms of positivity for rheumatoid factor or anti-citrullinated protein antibody at diagnosis. The prevalence of HLA-B27 was 14 of 114 (12.3%) and 8 of 11 (72.7%) in all patients with SpA patients and those with AS, respectively. Significantly fewer patients with LOSpA were HLA B27-positive than those with EOSpA. Although all patients with AS in the EOSpA group were HLA B27-positive, only 2 of 5 (40%) patients with AS in the LOSpA group were HLA B27 positive (data not shown).

**Table 2:**
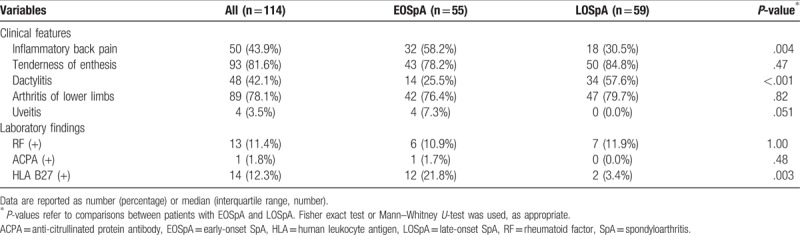
Clinical features and laboratory characteristics of patients with EOSpA and LOSpA (univariate analysis).

### 3.3 Fulfillment of SpA criteria

The Amor criteria were most commonly fulfilled in both groups (Table 3). None of the patients with LOSpA fulfilled the ASAS criteria for axSpA as these criteria do not apply to individuals over 45 years of age at onset. There were no significant differences in terms of the number of patients fulfilling each set of SpA criteria between the groups.

**Table 3:**
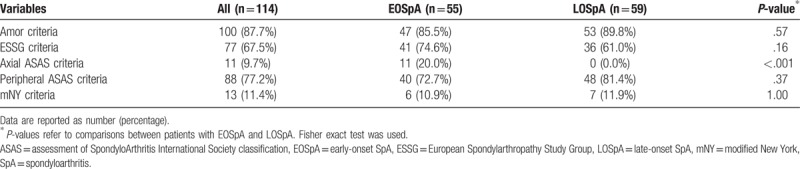
Patients fulfilling each set of SpA criteria (univariate analysis).

### 3.4 Imaging characteristics

There was no significant difference in the frequency of sacroiliitis on X-ray or MRI between the 2 groups (Table 4). Peripheral involvement by PDUS was evaluated in 47 patients with EOSpA and in 56 with LOSpA. On PDUS, articular synovitis was detected in 58 of 103 (56.3%) in all patients, and the median number of synovitis was 1.0. Synovitis of at least 1 joint was significantly more common in the LOSpA group than in the EOSpA group, particularly in the wrist. Bone erosion was not detected in PIP, MCP, and wrist joints of all patients except for 1 patient with PsA. Tenosynovitis or peritendinitis was detected in 67 of 103 (65.1%) in all patients, and the median number of tenosynovitis or peritendinitis was 1.0. Tenosynovitis or peritendinitis of at least 1 site was significantly more common in the LOSpA group than in the EOSpA group, particularly in the finger flexor tenosynovitis and wrist flexor tenosynovitis. Enthesitis was detected in 91 of 98 (92.9%) patients, and the median number of enthesitis was 2.0. In both EOSpA and LOSpA, enthesitis was the most frequent finding in PDUS. Enthesitis of finger collateral ligament and lateral collateral ligament were also significantly more prevalent in the LOSpA group.

**Table 4:**
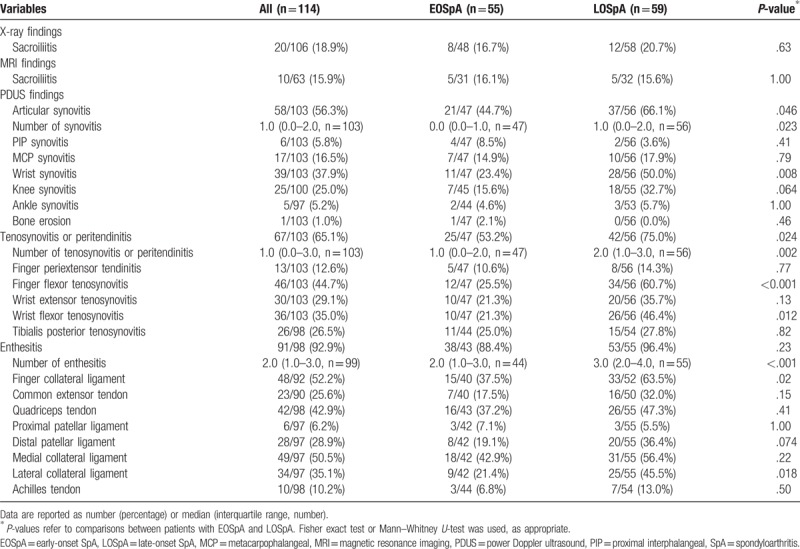
Imaging characteristics in patients with EOSpA and LOSpA (univariate analysis).

### 3.5 Independent factors associated with age at onset

We selected factors that varied significantly between the 2 groups in the univariate analysis and subjected them to multivariate logistic regression analysis. Only 1 factor was independently associated; IBP was more likely to be associated with the EOSpA group compared with the LOSpA group (Table 5).

**Table 5:**
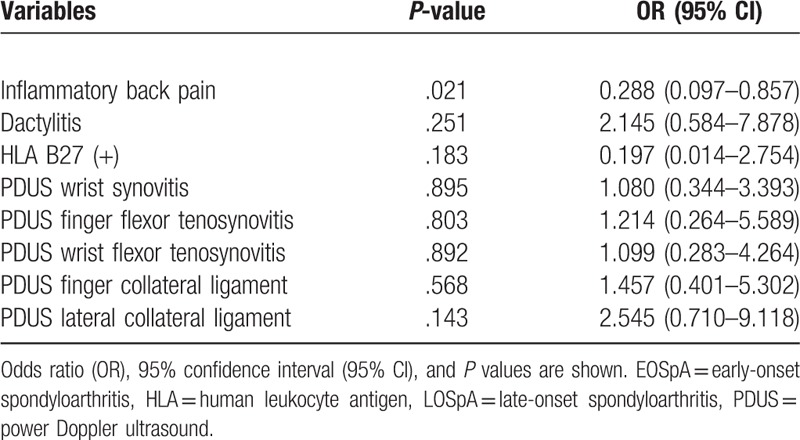
Multiple logistic regression analysis of selected variables in LOSpA compared with EOSpA.

## 4 Discussion

Our study clarified the clinical and laboratory differences between LOSpA and EOSpA in a retrospective cohort of Japanese patients. In addition, to the best of our knowledge, this is the first study to describe in detail the differences in PDUS findings in patients with EOSpA and LOSpA. Patients with LOSpA had shorter disease duration before diagnosis, lower prevalence of IBP, and HLA B27-positivity. In contrast, dactylitis was more common in the LOSpA group than in the EOSpA group, as were PDUS findings of articular synovitis (particularly wrist synovitis), tenosynovitis, peritendinitis (particularly finger and wrist flexor tenosynovitis), and enthesitis (particularly finger collateral ligament and lateral collateral ligament).

The prevalence of HLA B27 in Japan is much lower than that in other countries.^[10]^ Further, a previous study in Japan showed an entire of SpA prevalence of 0.04%, which is low compared with that in other countries.^[23]^ A study in Brazil showed that individuals with EOSpA had a significantly higher frequency of HLA B27-positivity than those with LOSpA, suggesting an association between HLA B27 and early-onset disease.^[14]^ A possible implication of our findings is that the extremely low prevalence of HLA B27 in Japan accounts for the lower prevalence of early-onset disease among Japanese patients with SpA. Moreover, the frequency of HLA B27-positivity is especially high in individuals with AS compared with other forms of SpA.^[24]^ In addition, patients with PsA with axial involvement reportedly had a significantly higher frequency of HLA B27-positivity than those with only peripheral involvement, which suggests an association between the presence of HLA B27 and axial involvement.^[25]^ Given these observations, Japanese patients with SpA probably have a lower frequency of AS and higher frequency of peripheral involvement because of the lower prevalence of HLA B27 in the Japanese population compared with that in other countries. Indeed, while the prevalence of AS is estimated to be between 0.1% and 1.4%,^[26]^ the estimated prevalence of AS is much lower in Japan (0.0065%).^[27]^ In our study, 8 of 11 patients (72.7%) with AS were HLA B27-positive. This frequency was comparable to that reported in the phase 3 trial of infliximab for AS in Japan (72.2%).^[28]^ However, the prevalence of HLA B27 was more than 90% among patients with primary AS in Western European countries.^[29]^ Although HLA B27-positivity in Japanese patients with AS has not been clarified, it may be lower than that in Western European countries.

Among 106 patients with SpA in Germany, 43.3% were diagnosed with uSpA, 30.2% with AS, and 14.2% with PsA, thus suggesting that uSpA is the most frequent type of SpA.^[30]^ A single-center cohort study in Japan showed that among patients with SpA (average age, 43.4 years), 40 (of 59, 67.8%) were classified as uSpA, 10 (16.9%) as AS, 6 (10.1%) as PsA, 2 (3.4%) as IBD-related arthritis, and 1 (1.7%) as ReA excluding palmoplantar pustular arthritis and SAPHO syndrome.^[23]^ Although average age was higher in our study, the proportion of SpA subgroups was similar. Consistent with the findings of our study, this study suggested a higher prevalence of uSpA in Japan compared with that in other countries. Late-onset uSpA appears to be comparatively more common than late-onset AS.^[31–33]^ It is important to recognize that even the elderly can develop SpA, particularly uSpA, despite the very low prevalence of HLA B27.

Our study showed that wrist synovitis, finger and wrist flexor tenosynovitis, and enthesitis of finger collateral ligament and lateral collateral ligament were more commonly detected on PDUS in the LOSpA group. PD signals of finger flexor tendon and finger collateral ligament are occasionally observed in dactylitis.^[34]^ A previous report from Brazil showed that patients with LOSpA had significantly fewer axial symptoms (ie, inflammatory buttock pain, radiographic sacroiliitis, alternating buttock pain, or hip involvement) and, conversely, a higher frequency of peripheral symptoms (ie, peripheral arthritis and dactylitis).^[14]^ Other reports have also suggested that peripheral involvement is more common in LOSpA.^[14,31–33]^ Late-onset PsA is also more associated with peripheral arthritis, with inflammatory edema of the joints.^[11,35–37]^ Consistent with a previous study,^[14]^ we found that patients with LOSpA had a shorter disease duration before diagnosis. This is possibly because of a higher frequency of peripheral symptoms in LOSpA with readily visible physical signs that can be more easily evaluated by physicians.^[14]^

The difficulty of definitively diagnosing SpA may also be a reason for an apparently low prevalence of the disease in Japan. A report from Canada showed that 11 of 35 patients initially considered to have fibromyalgia were eventually diagnosed with SpA.^[38]^ On the other hand, 50% of women with AS in another study also had fibromyalgia.^[39]^ This suggests that the 2 diagnoses may be confused with each other or that they may accompany one another. Late-onset uSpA might be misdiagnosed as RA, polymyalgia rheumatica (PMR), remitting seronegative symmetrical synovitis with pitting edema (RS3PE) syndrome, crystal-induced arthritis, or osteoarthritis.^[11]^ In a study of 20 patients with late-onset uSpA who were HLA B27-positive, 6 appeared with features mimicking RS3PE (ie, distal inflammation with edema on the dorsum of the feet and/or hands) and 3 had a PMR-like syndrome at onset.^[40]^ Therefore, it is important to distinguish other inflammatory rheumatic diseases from SpA. Clinical features, such as tenderness of enthesis, arthritis of lower limbs, and dactylitis are frequently detected in LOSpA. Thus, these findings are useful for distinguishing SpA from other rheumatic diseases. The use of PDUS early on to identify enthesitis and dactylitis may, thus, be advisable to facilitate an appropriate diagnosis in patients with peripheral symptoms.^[8]^

Although LOSpA has been considered rare,^[11]^ each form of SpA has been reported at the onset of the disease in older patients.^[12,14,41]^ In Japan, the aging rate is the highest than in other countries, and the population ratio of people over 65 years old was 26.6% in 2015. Our study was performed in an area with a super-aging population, where individuals aged >65 years account for 27.1% to 36.3% of the population.^[42]^ In the future, it is expected that the prevalence of LOSpA will increase as aging progresses. The different characteristics between LOSpA and EOSpA helps in not only making a correct diagnosis but also selecting the optimal treatment, thus avoiding side effects caused by unnecessary medication.

Our study has several limitations. First, there is no gold standard for the diagnosis of SpA.^[43]^ In our study, each patient was observed for ≥1 year by rheumatologists who performed comprehensive evaluations and excluded other rheumatic diseases. Because the patients were evaluated in routine clinical practice, PDUS findings may have introduced bias in the interpretation of clinical diagnosis. Moreover, we determined age at onset as the age when the patient presented the obvious symptoms associated with SpA. However, if the symptoms are insidious, it is possible that the patient may be grouped into LOSpA. Second, our study was a single-center retrospective study performed at a hospital in an area where the population is progressively aging, which might have caused selection bias. Because the sample size is small, there is a necessity to investigate by increasing the number in a nationwide multicenter research. Finally, there are no established standard values to evaluate disease activity in all forms encompassed by SpA. Therefore, we did not include disease activity as a variable in our study. Prospective multicenter studies are warranted to further compare the clinical profiles of EOSpA and LOSpA, including disease activity.

## 5 Conclusions

This is the first study to describe clinical differences between EOSpA and LOSpA in Japan. In particular, we noted differences in the PDUS findings between these 2 groups. We found that patients with LOSpA had a shorter disease duration before diagnosis, a lower prevalence of IBP or HLA B27-positivity, and a higher prevalence of dactylitis, and peripheral PDUS findings included articular synovitis (particularly wrist synovitis), tenosynovitis or peritendinitis (particularly finger and wrist flexor tenosynovitis), and enthesitis (particularly finger collateral ligament and lateral collateral ligament). It is important to distinguish LOSpA from other inflammatory diseases with peripheral involvement such as RA, PMR, RS3PE syndrome, and crystal-induced arthritis. Making a correct diagnosis of LOSpA facilitates selection of the optimal treatment and possibly reduces side effects caused by unnecessary medication.

## Acknowledgments

The authors wish to thank the patients and medical staff for their contribution to the study.

## Author contributions

KF and TK had full access to all of the data in the study and take responsibility for the integrity of the data and the accuracy of the data analysis. YE, KF, and TK helped with the study design. YE, KF, TK, AM, MM, TT, MU, and AK were responsible for acquisition of data. YE, KF, and TK were responsible for the analysis and interpretation of data. YE, KF, and TK prepared the manuscript. YE, KF, and TK performed statistical analysis. All authors read and approved the final manuscript.

**Supervision:** Akinari Mizokami, Masanobu Mine, Toshiaki Tsukada, Masataka Uetani, Atsushi Kawakami.

**Writing – original draft:** Yushiro Endo.

**Writing – review and editing:** Keita Fujikawa, Tomohiro Koga.
